# Virtual Versus Light Microscopy Usage among Students: A Systematic Review and Meta-Analytic Evidence in Medical Education

**DOI:** 10.3390/diagnostics13030558

**Published:** 2023-02-02

**Authors:** Sabyasachi Maity, Samal Nauhria, Narendra Nayak, Shreya Nauhria, Tamara Coffin, Jadzia Wray, Sepehr Haerianardakani, Ramsagar Sah, Andrew Spruce, Yujin Jeong, Mary C. Maj, Abhimanyu Sharma, Nicole Okpara, Chidubem J. Ike, Reetuparna Nath, Jack Nelson, Anil V. Parwani

**Affiliations:** 1 Department of Physiology, Neuroscience, and Behavioral Sciences, St. George’s University School of Medicine, St. George’s, Grenada; 2 Department of Pathology, St. Matthews University School of Medicine, Georgetown P.O. Box 30992, Cayman Islands; 3 Department of Microbiology, St. Matthews University School of Medicine, Georgetown P.O. Box 30992, Cayman Islands; 4 Department of Psychology, University of Leicester, Leicester LE1 7RH, UK; 5 Medical Student Research Institute, St. George’s University School of Medicine, St. George’s, Grenada; 6 Department of Public Health, Torrens University, Ultimo, Sydney, NSW 2007, Australia; 7 Department of Clinical Medicine, American University of Antigua, St. John’s, Antigua and Barbuda; 8 Department of Biochemistry, St. George’s University School of Medicine, St. George’s, Grenada; 9 Department of Pathology, Government Medical College, Jammu 180001, India; 10 Department of Education Service, St. George’s University, St. George’s, Grenada; 11 Medical Illustrator, The Centre for Biomedical Visualization, St. George’s University, St. George’s, Grenada; 12 Department of Pathology, Wexner Medical Center, The Ohio State University, Cooperative Human Tissue Network (CHTN) Midwestern Division, Columbus, OH 43210, USA

**Keywords:** digital pathology, dental students, education, medical students, medical school, virtual microscopy, whole-slide imaging, systematic review, meta-analyses

## Abstract

Background: The usage of whole-slide images has recently been gaining a foothold in medical education, training, and diagnosis. Objectives: The first objective of the current study was to compare academic performance on virtual microscopy (VM) and light microscopy (LM) for learning pathology, anatomy, and histology in medical and dental students during the COVID-19 period. The second objective was to gather insight into various applications and usage of such technology for medical education. Materials and methods: Using the keywords “virtual microscopy” or “light microscopy” or “digital microscopy” and “medical” and “dental” students, databases (PubMed, Embase, Scopus, Cochrane, CINAHL, and Google Scholar) were searched. Hand searching and snowballing were also employed for article searching. After extracting the relevant data based on inclusion and execution criteria, the qualitative data were used for the systematic review and quantitative data were used for meta-analysis. The Newcastle Ottawa Scale (NOS) scale was used to assess the quality of the included studies. Additionally, we registered our systematic review protocol in the prospective register of systematic reviews (PROSPERO) with registration number CRD42020205583. Results: A total of 39 studies met the criteria to be included in the systematic review. Overall, results indicated a preference for this technology and better academic scores. Qualitative analyses reported improved academic scores, ease of use, and enhanced collaboration amongst students as the top advantages, whereas technical issues were a disadvantage. The performance comparison of virtual versus light microscopy meta-analysis included 19 studies. Most (10/39) studies were from medical universities in the USA. VM was mainly used for teaching pathology courses (25/39) at medical schools (30/39). Dental schools (10/39) have also reported using VM for teaching microscopy. The COVID-19 pandemic was responsible for the transition to VM use in 17/39 studies. The pooled effect size of 19 studies significantly demonstrated higher exam performance (SMD: 1.36 [95% CI: 0.75, 1.96], *p* < 0.001) among the students who used VM for their learning. Students in the VM group demonstrated significantly higher exam performance than LM in pathology (SMD: 0.85 [95% CI: 0.26, 1.44], *p* < 0.01) and histopathology (SMD: 1.25 [95% CI: 0.71, 1.78], *p* < 0.001). For histology (SMD: 1.67 [95% CI: −0.05, 3.40], *p* = 0.06), the result was insignificant. The overall analysis of 15 studies assessing exam performance showed significantly higher performance for both medical (SMD: 1.42 [95% CI: 0.59, 2.25], *p* < 0.001) and dental students (SMD: 0.58 [95% CI: 0.58, 0.79], *p* < 0.001). Conclusions: The results of qualitative and quantitative analyses show that VM technology and digitization of glass slides enhance the teaching and learning of microscopic aspects of disease. Additionally, the COVID-19 global health crisis has produced many challenges to overcome from a macroscopic to microscopic scale, for which modern virtual technology is the solution. Therefore, medical educators worldwide should incorporate newer teaching technologies in the curriculum for the success of the coming generation of health-care professionals.

## 1. Introduction

The advent of the COVID-19 pandemic and physical distancing posed an unprecedented challenge to the world of medical education. How do you teach medicine, a human-centered subject that requires active interaction and engagement with people, without face-to-face contact? In response to this challenge, medical schools worldwide have implemented various changes such as online lectures and virtual classrooms in their education during the last two years to adapt to the new norm [1,2,3].

### 1.1. Whole-Slide Imaging (WSI)

Even before the pandemic, however, digital pathology using digital whole-slide imaging (WSI) was steadily gaining a foothold in medical education, training, and diagnosis [4,5]. Cumulative validations of the outstanding diagnostic concordance between WSI and glass-slide diagnoses prompted constant development and establishment of guidelines regarding WSI, thus progressively broadening the scope of its use [6,7]. Following the release of the guideline on the validation of WSI for diagnostic purposes by the Pathology and Laboratory Quality Center for Evidence-Based Guidelines of the College of American Pathologists (CAP) in 2013, WSI later gained approval from the Food and Drug Administration (FDA) for its use in primary diagnosis in 2017 and continues to be updated, the latest being the Guideline Update from the College of American Pathologists in Collaboration with the American Society for Clinical Pathology and the Association for Pathology Informatics in 2022 [6,8].

WSI technology is readily utilized by virtual microscopy (VM), a computerized conversion of light microscopy images in full resolution and their presentation over a computer network [9]. VM software can reproduce a digitized, high-resolution image of a traditional glass slide and allows the users to highlight, annotate, pan, and zoom. With the ease of use, added features, and reliability, interest in the exciting potential of VM continues to be on the rise [7,10,11].

### 1.2. Virtual Microscopy and COVID-19

Numerous literature reviews and meta-analyses reported the advantages of virtual microscopy before the global wave of digitization from the COVID-19 pandemic in 2019 [12,13,14,15]. Researchers have endlessly highlighted advantages of digital pathology using VM in medical practice [16,17,18,19]. These advantages include:

#### 1.2.1. General

- No risk of deterioration of staining quality or breakage of slides, no fading or stored slides, shorter sign-out time, access from any device, better flexibility, easy image sharing in clinical communication

#### 1.2.2. Telepathology

- Quick access, elimination of physical slide transfer, better availability of service for remote and understaffed areas

#### 1.2.3. Cost and Efficiency

- Better archiving, sharing, and easy retrieval; faster turnaround times, reduced cost of equipment, lab maintenance, and auxiliary techniques (less immunohistochemistry).

Advantages of VM use for medical educational are observed as well. Learners benefit from VM through remote teaching, multiple user access, the comfort of use amongst the modern “digital native” generation with prior computer knowledge, and better interaction between teachers and students by viewing the same image at the same time [17,20]. A meta-analysis by Wilson et al. also found that learners prefer VM to conventional light microscopy as well [13].

One notable advantage of using VM worth acknowledging is the benefit of access to slide images without restricting time and space. This unique characteristic of VM came into the spotlight upon facing the lockdowns during the coronavirus global health crisis. To ensure undisrupted quality education for students, lecturers adjusted their teaching methods to social distancing and disease-prevention regulations accordingly. In addition, the massive shift in medical education towards remote learning and digitization of the learning materials granted researchers an abundant opportunity and data to investigate VM more deeply. With the already known benefits of using digital slides, additional positive effects such as self-paced learning, improved tissue recognition due to better access to slides, improved understanding, and better academic performance have been reported during COVID-19 lockdown-adapted online classes [2,21,22].

In this review, we aim to compare the academic performance of medical students by using VM technology to learn the microscopic aspect of the disease. In addition, this study intends to include recent data on VM and WSI to present the most updated synthesis on VM and to explore any differences in usage, benefits, and drawbacks of VM that may have been newly discovered during the COVID-19 era.

## 2. Materials and Methods

This review reports the systematic findings according to the Preferred Reporting Items for Systematic Reviews and Meta-Analysis (PRISMA) guidelines [23]. The systematic review protocol was registered in the prospective register of systematic reviews (PROSPERO; https://www.crd.york.ac.uk/prospero (accessed on 21 September 2020)) with registration number CRD42020205583.

The review questions were “Does virtual or digital microscopy enhance student exam performance?” along with secondary qualitative assessment: “Is virtual microscopy a reliable and a better method for teaching and learning in medical education?” and “What are the student preferences for this newer technology?”

### 2.1. Literature Review

One author (NN) performed a literature search to identify if any systematic reviews were available or protocols registered as to our study objective. We identified three similar reviews [13,14,24]. However, these reviews had major limitations, such as not including studies that measured the efficacy of VM during or after the COVID-19 pandemic period. One of these previous studies included both medical students and pathology residents [24]. Its literature search was performed in a limited number of databases and failed to report comprehensive search criteria. Furthermore, these studies had narrow selection criteria, including only the pathology course at medical school, despite existing papers demonstrating VM or LM use in cytopathology, anatomy, histology, or hematopathology courses in medical, dental, and veterinary schools.

### 2.2. Eligibility Criteria

Only original research articles assessing the performance of LM and VM through the process of any data type—academic scores, student feedback, questionnaires, and surveys—were included for this review. Additionally, we included articles assessing the performance or perception of medical or dental students using VM or traditional LM. Articles were included irrespective of use for pathology, histology, anatomy or histopathology. The meta-analysis included comparative studies of LM versus VM or crossover studies. Studies with data on the students’ performance measured as a percentage or score on a definitive scale and clear mention of method of evaluating students’ perceptions were included. Studies published in English were included (or others if the translation in English was available).

Studies mentioning a VM resource or description of the technology used in medical or dental schools were also included. Along with this information, studies describing VM use due to the transition toward online teaching during COVID-19 were included.

### 2.3. Exclusion Criteria

Studies that described VM used for pathological diagnosis or involving perceptions of pathology residents were excluded from this review. Literature reviews (systematic, meta-analyses, narrative), editorial letters, book chapters, and case reports were excluded. Publications in which the modality of WSI was unclear/unspecified or no data (qualitative or quantitative) in the form of survey or comparison were available were also excluded.

### 2.4. Search Criteria and Database

A comprehensive database search was performed on 15 December 2021 and again on 15 March 2022 (to include updates) from the date of inception in Scopus, PubMed, CINAHL, Web of Science, Embase, Cochrane Library and Google Scholar. Various search terms such as “virtual microscopy/microscop*,” “digital microscopy/microscop*,” “virtual slides,” “whole slide imaging,” “students,” and “medical education” in combinations of Boolean operators and truncation were used to ensure comprehensive inclusion of relevant articles. Search criteria were adjusted to the selected database. In addition, we manually searched recent reviews or eligible studies to identify any potential studies.

### 2.5. Article Screening and Eligibility Evaluation

For a fair screening process, two teams (SM, SN, and ShN; JW, TC, and SH) of researchers independently performed title and abstract screening based on study inclusion criteria. In addition, we performed a full-text analysis if the potentially relevant article’s abstract did not contain sufficient information. The inclusion and exclusion criteria were used to select the eligible studies and access the full-text articles. Zotero software was used as the reference manager to import the search results from the database and exclude duplicates [25]. Google Sheets was used to screen the articles and register a primary reason for exclusion. Disagreements were resolved by collective discussion involving both teams, which ensured that appropriate publications were selected according to the eligibility criteria.

### 2.6. Extraction of Qualitative and Quantitative Data

One author (SM) independently extracted the available data from the eligible studies, followed by the second author (SN) reviewing the extracted data. Finally, we designed a standardized data collection Google sheet to organize the qualitative and quantitative data.

For each selected study, the following information was extracted (when available): year and country of publication, which variable was analyzed (performance, perception or both), number of participants, students’ educational level, type of equipment and software used to assess WSI, types of workstation, digital slide accessibility, equipment training, LM availability and its specification, number and Scope of used samples, and how the students’ performance and/or perception were assessed and their results. The outcome of interest for this meta-analysis was focused on estimating overall exam performance based on discipline and subject.

### 2.7. Quality Assessment

Two authors independently (SM and RN) used the original version of the Newcastle Ottawa Scale (NOS) for the quality appraisal of the included studies [26]. The NOS scale is a star-based system that evaluates the study based on three major perspectives: the selection of the study groups, comparability of the groups, and the ascertainment of either the exposure or outcome of interest for non-RCTs. For case–control studies, a study was awarded a maximum of one star for each numbered item within the selection and exposure categories. A maximum of two stars were given for comparability. For cohort studies, a study was awarded a maximum of one star for each numbered item within the selection and outcome categories. For comparability, a maximum of two stars were given (see Supplementary File S1). Finally, each study was categorized as good, fair or poor quality. A subgroup quantitative analysis of the studies was done after classifying them as good, fair or poor quality according to Agency for Health Research and Quality (AHRQ) standards (see Supplementary File S2). Any discrepancies were resolved by discussion with the third reviewer (SN).

### 2.8. Statistical Analyses

The Google sheet was cleaned and organized to conduct qualitative and quantitative analysis. Qualitative results were organized and included in the systematic review, whereas the quantitative data were analyzed further to estimate the overall better educational technique (VM versus LM). Review Manager version 5.4 calculated mean differences, pooled effect size, and heterogeneity. Only the studies with data on comparative exam scores went to the quantitative analytical stage (meta-analyses). Since the overall analysis demonstrated considerable heterogeneity, the random effect model to generate forest plots and publication bias was used. The choice of a random effect model was made due to the heterogeneity that was observed for different countries, different year of study, different faculty, different discipline, different teaching technique, different technical setup, and pre/COVID-19. Included studies used different scales to measure the same outcome, i.e., the units for the outcome of interest were different across studies. For such cases, the mean differences (MD) cannot be directly pooled and analyzed. Thus, MD was divided by the respective standard deviations (SDs) to yield a statistic known as the standardized mean difference (SMD) [27]. Therefore, the extracted data were computed and organized as continuous data followed by an inverse variance analysis method to estimate the SMD and 95% confidence interval. The heterogeneity was assessed using Higgins square I^2^ or Q-statistic. I^2^ can be interpreted as minimal (0–40%), moderate (30–60%), substantial (50–90%) and considerable (75–100%) [28]. Begg and Mazumdar’s rank correlation and Egger’s test were used to confirm the publication bias [29]. Subgroup analyses according to the subject (pathology, histology or histopathology) and faculty (medical or dental) were also performed.

In the qualitative data review of included articles, themes that referred to the applications, advantages, and disadvantages of VM were identified. In addition, perceptions, surveys, or questionnaire data related to student experiences with VM were extracted from the Google sheet.

## 3. Results

### 3.1. Search Results and Study Characteristics

**Figure 1:**
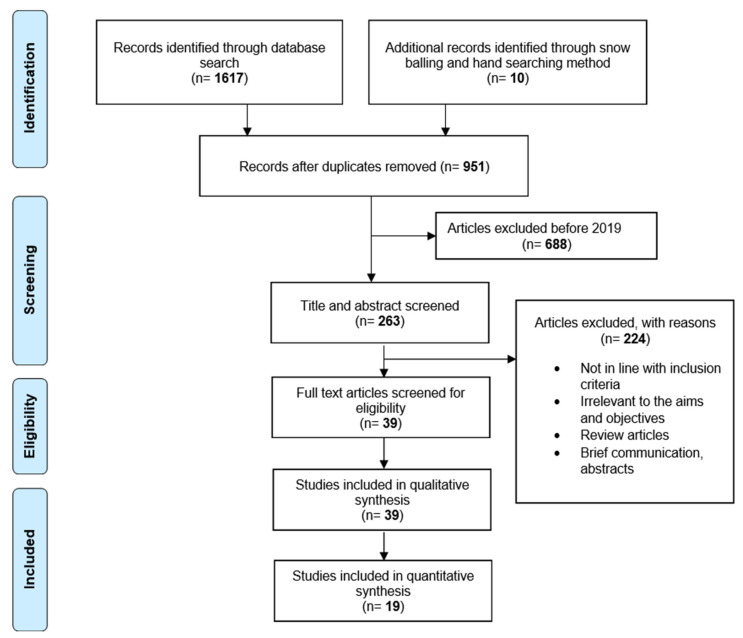
Included 19 studies (PRISMA flow diagram).

A total of 1627 studies were identified from the selected database search. After removal of 676 duplicates, there were 951 eligible studies, of which those conducted before year 2019 were further excluded. Thus, a final number of 263 articles were screened for title and abstract and 39 full-text articles were reviewed to be included in the systematic review. The meta-analysis of the performance comparison of VM versus LM included 19 studies (see flowchart in Figure 1).

The included articles were published from 2019 to 2022, and originated from North America, South America, Europe, Australia, the United Kingdom and Asia. Most (10/39) were from medical universities in the USA. VM was mostly used for teaching pathology (25/39) at medical schools (30/39). Dental schools (10/39) also reported using VM for teaching microscopy. The most commonly used VM software reported by the studies (6/39) was Aperio ImageScope [30]. Only three studies in this review collected data using a randomized controlled trial protocol, whereas most collected data were based on group performance comparison without randomization. COVID-19 was responsible for transition to VM use in 17/39 studies. A detailed synthesis of included studies in this review is provided in Table 1.

Using Table 1, we performed thematic analyses for the advantages/disadvantages reported by various studies on using VM or LM. Thematic analyses revealed various codes describing the advantages and disadvantages from the included articles (Supplementary Table S1). The top themes highlighting advantages that emerged were improvements in academic performance of the students, ease of use of VM, a positive student perception and acceptability of VM, and enhanced cooperation and student collaboration. In addition, one of the generated themes highlighted a positive impact on the teaching faculty.

Results from various studies revealed significantly improved academic test scores [20,31,32,36,37,38,46,48,50,52,53,58,62,63] along with improvements in the medical knowledge of students [22,34,38,44,45,46,48,55,58,61,63,64,67].

Development of diagnostic and practical skills during laboratory sessions was an important finding [33,39,48,53,56,58,59,64]. Studies also reported that VM promotes self-directed learning [31,35,38,41,48,58,59,64] and thus an overall better method of learning for exam preparation for the students [32,38,52,53,56].

Accessibility to the slide images outside classroom [35,44,47,48,49,52,53,54,59,64,67], ability to annotate slides [20,35] and availability of ample free resources led to a more efficient and feasible method of learning [20,34,40,41,44,48,54,67].

An overall positive acceptance for VM with a higher student satisfaction for VM-based teaching in addition to increased levels of subject interest was another reported advantage [20,22,35,36,37,39,41,47,49,50,53,54,55,59,60,64,66,67].

Improved student and faculty rapport [33,34,42,45,51,52,56,60] as well as better cooperation and participation amongst students was another interesting finding [20,31,32,33,34,36,43,52,57,60,61,66,67].

The teaching faculty also reported higher levels of satisfaction with VM use [20,35,42,57,59] and also reported VM as a time-saving and cost-effective teaching method [20,31,32,33,35,36,42,57,67].

Technical and internet issues while accessing the slides were the main disadvantages [22,33,38,41,44,57,67]. Few studies reported less interaction and impaired social connections along with a lack of faculty feedback as main disadvantages [22,41,44].

### 3.2. Quality Assessment of Included Studies Using NOS

The final included articles were predominantly of cross-sectional design, and thus an adapted version of the NOS scale was applied for quality assessment of cross-sectional studies. Others were evaluated using the original NOS scale. To evaluate each study, a n asterisk was assigned to any of the fulfilled criteria in the selected scale parameter. Table 2 represents the summary of quality assessment using the NOS scale for cohort studies.

Table 3 represents the summary of quality assessment using the NOS scale for cross-sectional studies (total nine articles).

Table 4 represents the summary of quality assessment using the NOS scale for randomized controlled studies (total three articles).

Table 5 represents the summary of quality assessment using the NOS scale for case–control studies (total seven articles).

**Figure 2:**
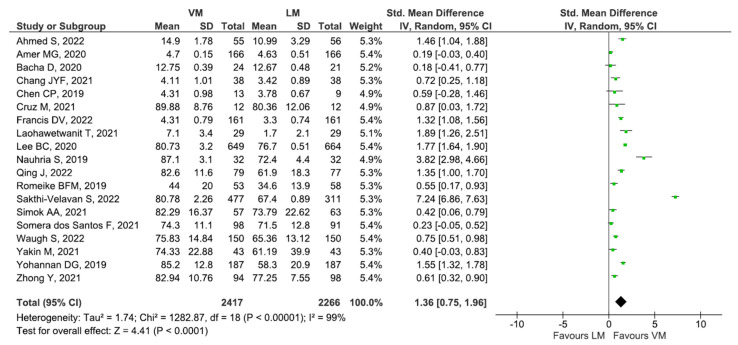
Pooled effect size of 19 studies [20,31,32,33,34,35,37,38,39,44,45,50,52,57,58,59,60,63,64].

The pooled effect size of 19 studies significantly demonstrated higher exam performance (SMD: 1.36 [95% CI: 0.75, 1.96], *p* < 0.001) among the students who studied by VM method than the LM method with considerable heterogeneity (I^2^: 100%, *p*-value <0.001) as shown in Figure 2.

**Figure 3:**
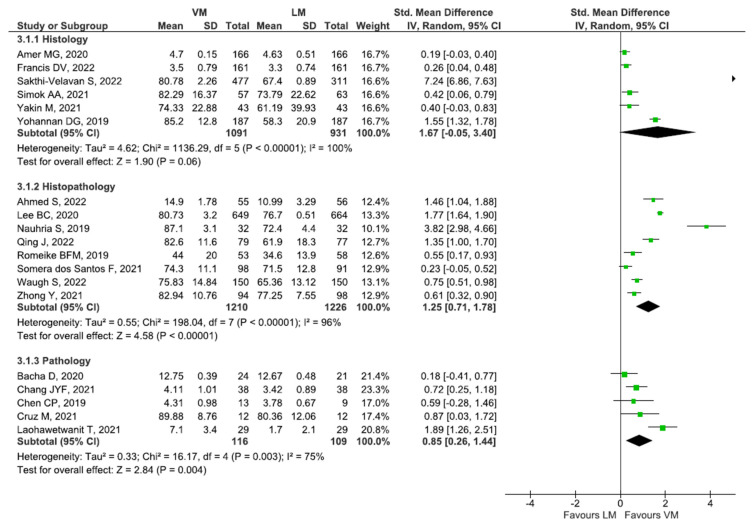
Analysis of studies assessing subject-wise exam performance of students [20,31,32,33,34,35,37,38,39,44,45,50,52,57,58,59,60,63,64].

Students in the VM group demonstrated significantly higher exam performance than LM in pathology (SMD: 0.85 [95% CI: 0.26, 1.44], *p* < 0.01) and histopathology (SMD: 1.25 [95% CI: 0.71, 1.78], *p* < 0.001). For histology (SMD: 1.67 [95% CI: −0.05, 3.40], *p* = 0.06), the result was insignificant (Figure 3).

**Figure 4:**
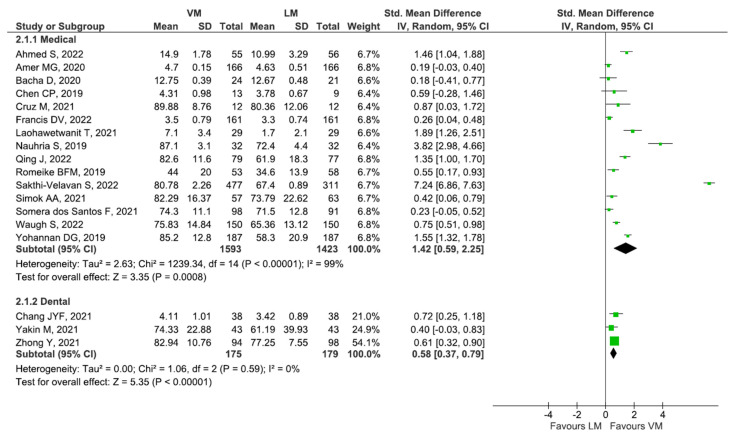
Overall analysis of 15 studies assessing exam performance among medical students [20,31,32,33,35,44,45,50,52,57,59,60,63,64].

The overall analysis of 15 studies assessing exam performance showed significantly higher performance for both medical (SMD: 1.42 [95% CI: 0.59, 2.25], *p* < 0.001) and dental students (SMD: 0.58 [95% CI: 0.58, 0.79], *p* < 0.001) under VM learning than the conventional method (Figure 4).

**Figure 5:**
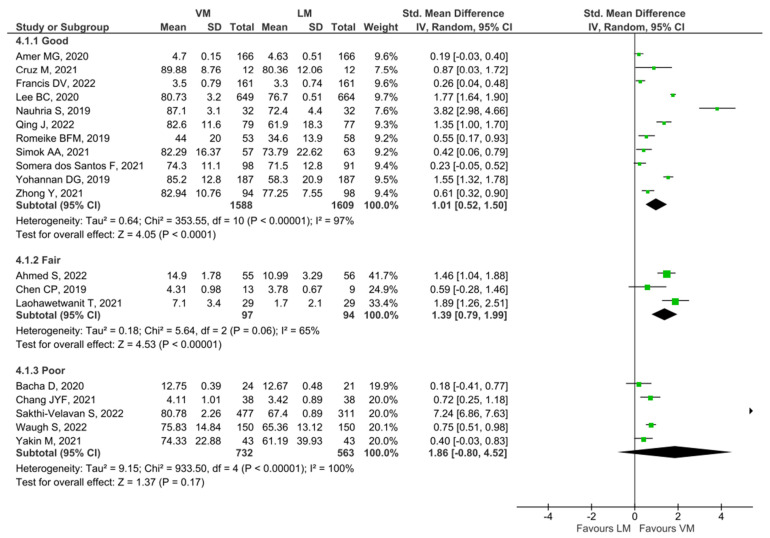
Analysis of studies based on NOS scale quality [20,31,32,33,34,35,37,38,39,44,45,50,52,57,58,59,60,63,64].

A subgroup analysis on studies of low risk or bias compared to higher risk of bias was also performed based on the results of the NOS scale (Figure 5).

In sum, 11/19 studies were categorized as good quality, whereas 3/19 were fair and 5/19 were of poor quality. The result showed a clear significance for the “good” subgroup (SMD: 1.01 [95% CI: 0.52, 1.50], *p* < 0.001) as well as the “fair” subgroup (SMD: 1.39 [95% CI: 0.79, 1.99], *p* < 0.001). The result was not significant for the “poor” rated studies (SMD: 1.86 [95% CI: −0.80, 4.52], *p* = 0.17).

**Figure 6:**
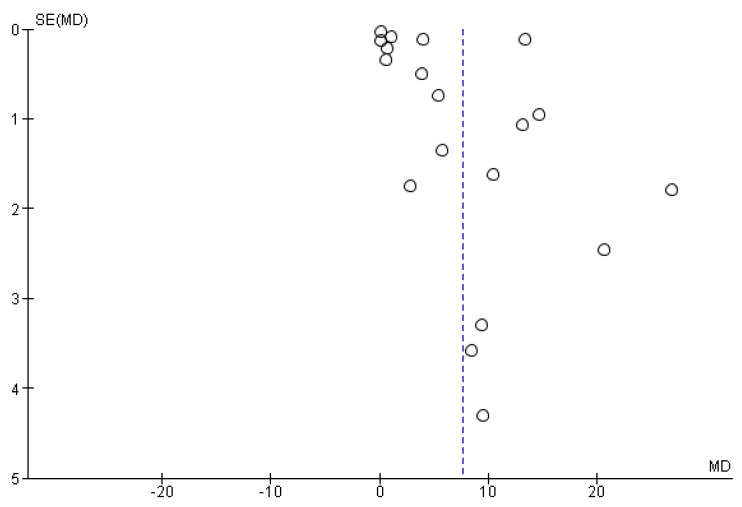
Publication bias (funnel plot).

The studies in the funnel plot are distributed asymmetrically, which suggests publication bias. Begg’s and Mazumdar’s for rank correlation have a *p*-value of 0.19, suggesting publication bias. Eggers test for a regression intercept of 10.36 resulted in *p*-value (one-tailed) of 0.06, which confirms the presence of publication bias (Figure 6).

## 4. Discussion

In this review article, the authors compared the utility of VM for teaching medical subjects in medical and dental schools. The results of our qualitative and quantitative analyses show a comparison of student performance after using VM technology. The digitization of glass slides undoubtedly enhances the ease of teaching and learning of microscopic aspects of disease. Additionally, the COVID-19 global health crisis has produced many challenges to overcome from a macroscopic to microscopic scale, for which modern virtual technology has been the solution [1].

The authors performed a systematic review considering how to remove existing literature limitations. A well-designed study criterion to include more studies, including the articles published during COVID-19 pandemic to analyze available evidence on the usage of VM for the learning process for medical and dental school learners compared to the traditional LM, was developed.

The results from the systematic review clearly show a preference for using VM. In contrast, the meta-analysis results statistically prove that overall student performance on the examination is better when using such technology for learning.

As the use of virtual learning platforms and virtual meeting spaces proliferated, educators of undergraduate and preclinical sciences adapted to using VM with remote students [2]. The use of VM in education is not unprecedented, of course, but its application and scope has significantly expanded over the last two years [21,32,36,68,69]. Much like modern students are more likely to have experience editing a digital photograph than developing an analogue photograph, the technology offers a more intuitive experience to the novice user. Clinical medicine, too, has shown VM to be a ready solution for histopathology diagnosis, supported by double-blind evidence of no inferiority to traditional modalities of diagnosis [70,71,72].

Various researchers around the globe have highlighted the advantages of such digital technologies when used for histopathology diagnosis, including diagnosis for such specialties as dermatology, neurology, gastrointestinal, cancer pathology and hematological diagnosis [73,74,75,76,77,78]. The advent of newer artificial intelligence (AI) and machine learning technologies that are being embedded into digital pathology and VM software have extended a pathologist’s diagnostic capabilities beyond the scope of the tissue section on a glass slide [79].

The newer applications and ever-increasing usage of VM software and WSI systems propel the need to integrate such technologies for medical education, especially at the undergraduate level, as students may encounter such technology during their residency years and during medical practice.

VM has gained increasing interest in the last four decades. The benefits over traditional LM include the practical, such as storage and maintenance of slides on a hard drive with backup, and the user experience, where an entire classroom can work with a single slide. For many years, however, this resource had severe limitations due to limited data storage and image magnification technology. Using WSI technology, starting in the early 2000s, VM has allowed the user to choose the magnification of the image with the stroke of a mouse and with less technical skill than traditional LM [9,17].

Results of this systematic review highlighted findings with a focus on advantages of VM use for both students and medical teachers. Most of the included articles mentioned various advantages, such as ease of use of digital slides. Easy access and constant availability with online access were the top advantages, whereas cost of implementation was the most discussed disadvantage of VM. The current COVID-19 pandemic has clearly given a boost to the field, so more robust real-world data from larger-scale VM implementations can be expected soon.

While students have largely returned to in-person learning in the pandemic’s third year, many of the innovations and remote learning adaptations of the pandemic are being integrated rather than discarded [80]. Prevailing tides of change in the digital era were already moving academic histopathology away from traditional LM in favor of a modern approach [19]. Digitizing the workspace has been a welcome improvement for learners. In one survey of pathology students utilizing VM in a remote clerkship, respondents reported greater interest and understanding of the material [22]. While some larger organizations have been able to produce and maintain their own VM database, smaller organizations have been able to benefit from the free access many institutions have offered.

An interesting aspect of the review findings in this study were the areas of VM use in medical education. Various VM-based learning activities have been employed by medical educators. Such activities include active learning activities such as group discussions, collaborative discussions, podcasts and clinical case discussions. In this review, the results show an overall positive impact of VM in a digitized learning environment and evidence indicates high acceptability and adaptability by medical learners.

Educators at the University of Eastern Finland initiated a curricular reform for histology education focused on development of a student-centered WSI platform [66]. A “gamification” histology learning model was developed that is based on incorporation of game mechanics and game theory into education [81,82].

Introduction of such a learning system into dental and medical histology courses stimulated learning and improved student satisfaction [66].

Another interesting application of VM by Sakthi-Velavan et al. involved the blending of histology content using podcasts into an integrated curriculum [32]. VM-based podcasts are narrative recordings of digital histology images. Results of the study showed a positive association between podcast viewing and improved overall class performance. The students reported a better learning experience after using the podcast-based VM. The findings align with the current review and previous studies on exploring the effectiveness of VM podcast-based teaching in medical schools [83].

During the COVID-19 pandemic, educators were forced to transition to an online distance learning pedagogy. Westfälische-Wilhelms-University, Germany and the University of California San Francisco are a couple of many such universities that successfully transitioned the entire VM-based courses to a completely online distance learning histology and pathology curriculum. Researchers at these universities used customized VM university databases and reported that the implementation of a curricular histology course in an online format was technically realizable, effective and well accepted among students. While distance learning models are insufficient for career progression in pathology, VM can still be adapted to enhance collaboration and microscopic learning of disease [40,41].

High costs of WSI scanners and VM software pose a significant challenge for adoption of digital microscopy in medical schools. Ample alternative options are available for implementation of VM for teaching and learning microscopy. Such resources include free online websites or cloud-based servers that can be accessed via the internet freely for educational purpose. Results from the analysis of the studies included in this review highlight the use of such free websites and cloud-based servers for VM resources [20,31,36,38,43].

One of the most used VM apps in this review was Aperio ImageScope, which allows viewing online or local network WSI [30]. The Biolucida viewer [84] is another such VM viewer that connects to the digital slide cloud library at the University of Iowa and can be viewed freely worldwide without any cost involved. The University of Michigan Virtual Slide Box [85] and Virtual Pathology at the University of Leeds [86] offer many WS images that can be viewed over the internet by any available web browser.

Another interesting aspect of VM implementation in medical education is the development of competence in students. American accreditation agencies such as the Accreditation Council for Graduate Medical Education (ACGME) [87] and Liaison Committee on Medical Education (LCME) [88] have outlined core skills that need to be addressed by medical schools to meet the required educational standards, including medical knowledge, patient care, communication skills, professionalism, lifelong learning and social context of health care [89]. Due to the accreditation body requirements along with the ongoing transition towards remote or distance learning, implementation of VM can help in addressing such competence and ensuring development of competent physicians for the society [9,20].

### Limitations of this Review

A significant limitation of our study is the presence of a considerable level of heterogeneity. This could be due to the methodological (differences in study design, risk of bias, etc.) and statistical (variation in intervention effects or results) differences from the diverse geographical population with different cultures. For example, Lee et al. (2020) reported academic scores as percentages to compare LM and VM groups. However, Chen et al. (2019) reported academic scores on a five point scale to compare the academic improvement by using the VM. The quality of published articles can be further improved by standardizing of the research design and methodology for such an educational intervention. The NOS scale resulted in only four studies with a high score (six or seven stars) and 15 studies had a score above three stars. A total of 24 out of 39 studies had poor research design according to the NOS scale. As most educators and medical schools around the world use a wide spectrum of teaching methods along with diverse curricular designs, results for educational intervention impact will continue to be heterogeneous.

Nonetheless, this meta-analysis provides strong evidence that students prefer VM over LM (although it cannot be replaced completely) and exam performance also increased by using VM. The heterogeneity of results and different outcomes posed could be resolved in the future by introducing more subgroup analysis that would still need some homogeneous data set to work on.

## 5. Conclusions

This review highlights various advantages of VM compared to traditional LM for medical education. Most studies in this meta-analysis were pilot projects and first-time implementation of digital technology at various medical universities. Globally, VM and WSI technology have undoubtedly reshaped pathology teaching and learning in medical and dental schools. Use of VM in medical education has provided a venue for stimulated learning, improved student satisfaction and an overall better learning experience. Easy access to educational content and constant availability with online access are amongst the top advantages, whereas cost of implementation and access to the internet are still the most discussed disadvantages of such technology.

Availability of numerous free online VM resources has fueled global access to educational materials geared towards learning microscopy of normal tissue and pathological features of various human diseases. The ongoing COVID-19 pandemic has further fueled the need for digitization of teaching methods, particularly increased use of VM in medical education worldwide. As much of the current work on VM usage outcomes is from early technology implementation, a certain degree of enthusiastic bias in favor of VM is inevitable.

## Figures and Tables

**Table 1:** Characteristics of all included studies.

Author and Year	University and Location	Course Subject/Medical or Dental	Study Design	Total Participants	VM Setup Used	Conclusion/Results
Waugh S, 2022 [31]	Griffith University, Australia	Histopathology, medical students	Observational case–control study	150	BEST slice cloud-based library	A thematic analysis of the qualitative comments strongly indicated that online histopathology teaching was instrumental, more comfortable to engage in and better structured compared to face-to-face teaching. Compared to the prior cohort completing the same curriculum the mean overall mark was significantly improved.
Sakthi-Velavan S, 2022 [32]	Marian University College of Osteopathic Medicine, USA	Histology, medical students	Observational case–control study	477	VM podcast	Most students indicated that the podcasts enabled more efficient study time and improved their confidence in the histology content on examinations. A summary of students’ feedback and academic performance supported that integration of the VMPs into Histology teaching improved the learning experience. The findings align with previous studies on the effectiveness of multimedia-based teaching in histology laboratory modules. There was a significant difference between the average histology performance of earlier classes that did not have access to the VMPs versus the average performance of the classes that had access to the VMPs.
Ahmed S, 2022 [33]	Shifa College of Medicine, Pakistan	Pathology, 3rd-year medical students	Randomized crossover control study	111	Not specified	Evidence showed that the microscopic practical skills achieved by virtual microscopy are comparable to or even better than those achieved by light microscopy.
Qing J, 2022 [34]	Wuhan University, China	Histopathology, dental students	Observational case–control study	156	NanoZoomer Digital Pathology sofware	Study compared results of assignments and exams between VM group and LM group and a questionnaire survey was used to collect feedback. Results showed an increase laboratory final test grades increased and the feedback of the questionnaire was positive, indicating that students were satisfied with the system. This study concluded that VM is an efficient and feasible teaching technology and improves students’ academic performance.
Francis DV, 2022 [35]	Christian Medical College, Vellore, India	Histology, medical students	Observational case–control study	100 (cohort one), 99 (cohort 2)	VM software-Open Microscopy Environment Remote Objects (OMERO), University of Dundee, UK. WSI scanner-Digiscan (https://digiscan.co.in/)	Majority students were reported to be enthusiastic about using VM. Some of the benefits of VM as cited by the students were the ease of usage, annotations, the superior quality of images, accessibility to slides outside of lab time, in class internet access to additional learning material, promotion of self-learning and efficient use of their study time. Performance score analysis showed a statistically significant improvement of grades in the VM arm.
Nikas IP, 2021 [36]	School of Medicine, European University, Cyprus	Histology and pathology, medical students	Cross-sectional surveys	173	Websites e.g., Michigan Histology and Virtual Microscopy Learning Resources	Both histology and pathology online delivery was well-accepted by most medical students. Pathology students and students with high final examination scores perceived their virtual education more favorably.
Zhong Y, 2021 [37]	Nanjing Medical University, China	Histopathology, dental students	Comparative cross-sectional	192	NanoZoomer Digital Pathology	The mean scores of the online group (VM) were significantly higher than those of the traditional group (LM). Furthermore, both remote learning and virtual microscopy courses were well accepted by students according to the questionnaire.
Yakin M, 2021 [38]	Adelaide Dental School, University of Adelaide, Australia	Histology, year 1 and year 3, dental students	Comparative prospective cohort study	43	Biomedical Education Skills and Training network (www.best.edu.au)	Students obtained significantly higher scores in experimental exam questions than control exam questions. A significantly larger number of students perceived that the adaptive lessons improved their knowledge of the subject.
Chang JYF, 2021 [39]	National Taiwan University, Taiwan	Oral pathology, dental students	Comparative cross-sectional	38	Dot-slide system developed by Soft Imaging System GmbH (Olympus Deutschland GmbH, Hamburg, Germany)	Results showed a significantly higher acceptance rate and a significantly better histopathological diagnosis ability among dental students using the virtual slide learning than those using the glass-slide learning for the oral pathology laboratory course.VM with digitized virtual slides may gradually replace the real microscopy with glass slides for the learning of oral pathology laboratory course.
Darici D, 2021 [40]	Westfälische-Wilhelms-University, Germany	Histology, preclinical medical students	Cross-sectional cohort study	400	Custom histology software-Virtuelle Mikroskopie	The study concluded that the implementation of a curricular histology course in an online-format is technically realizable, effective and well accepted among students. The study also reported that availability and prior experience with digitized specimen in VM facilitates transition into an online-only setting.
Tanaka KS, 2021 [41]	University of California, USA	Pathology, fourth year medical students	Cross-sectional cohort study	37	Custom UCSF digital library	End-of-rotation data showed the remote pathology course performed well when compared to the traditional in-person pathology elective. Core strengths highlighted in this study include a high educational value, flexibility of content and schedule, organization, tailoring to an individual’s learning goals and a positive education environment. Drawbacks were the inability to gross surgical specimens, inadequate observation or feedback about students’ skills, and impaired social connections.
Tauber Z, 2021 [42]	Palacky University, Czech Republic	Histology, dental students	Structured questionnaire	82 Dentistry, 192 General medicine	Not mentioned	All students in this study indicated that they prefer the use of VM or the combination of VM together with the examination of glass mounted specimens by microscope.
Guiter GE, 2021 [43]	Weill Cornell Medicine, Qatar	Pathology, medical students	Cross-sectional surveys	29	University of Leeds’ Virtual Pathology Library	Students conveyed high levels of satisfaction about the elective’s overall quality, their pathology learning and online interactions, with minimal challenges related to the remote nature of the course.
Somera dos Santos F, 2021 [44]	Ribeirao Preto Medical School, Brazil	Histology, medical students	Cross-sectional Cohort study	189	NanoZoomer S60 digital whole slide scanner	The study reported positive subjective feedback related to handling, suitability, learning effectiveness, and pleasure using the tools for VM. Although no statistically significant differences were found between groups for academic performance, VM proved to be adequate to the Brazilian medical education in light of Brazilian social contexts and COVID-19 pandemic.
Cruz M, 2021 [45]	Cooper Medical School of Rowan University, USA	Pathology, medical students	Comparative cross-sectional	44	Web-basedprogram (Aperio)	VM could help first- and second-year medical students understand case-based scenarios and clinical pathology more deeply than photomicrographs, particularly with direct faculty support for navigating virtual slides. Participation in and completion of pathology-VM learning modules enhances student learning of pathology-related topics.
Sharma R, 2021 [46]	School of Medicine, University of Texas Health San Antonio, USA	Histopathology, medical students	Cross-sectional cohort study	215 (MS1); 207 (MS2)	Not specified	Majority students agreed that the VM helped in their learning. Students performed better in module examinations in 2020 than in the previous years.
White MJ, 2021 [47]	Johns Hopkins University, USA	Pathology, medical students	Cross-sectional surveys	43	Leica Aperio AT and Roche iScan HT	Most students provided positive objective feedback related to VM use.
Lakhtakia R, 2021 [48]	Mohammed Bin Rashid University of Medicine and Health Sciences, UAE	Pathology, medical students	Cross-sectional surveys	49	Cirdan PathXL Tutor, Lisburn, Ireland	VM usage was reported as a user-friendly resource that helped students develop a strong clinical foundation and clinico-pathological correlation. High student attendance and improved assessment scores on critical thinking were observed. Easy access was a significant student-centric advantage reported by this study.
Liu Q, 2021 [49]	Shandong First Medical University, China	Histology, embryology and pathology, medical students	Observational case–control study	512	Medical Morphology Digital Teaching System	With regard to the teaching performance of VM based teaching, students demonstrated a high degree of satisfaction. Majority students achieved high scores in the web-based learning group than in the offline learning control group.
Simok AA, 2021 [50]	Universiti Sains, Malaysia	Histology, medical students	Randomized control study	120	Pannoramic viewer VM software by 3DHISTECH Ltd.	The VM group had a significantly higher satisfaction score towards the learning tool than the LM group. The knowledge acquisition of the VM group was equal to the LM group as they were shown to have a similar improvement in the test scores, comprehension level and learning ability. The study revealed a significant improvement in test scores for VM.
Manou E, 2021 [51]	National and Kapodistrian University of Athens, Greece	Pathology, medical students	Observational cohort study	91	e-learning platform HIPON (HistoPathology Online)	The study concluded that further research to enhance understanding of the aspects of the e-learning environment towards the formulation of policies for higher-quality education is needed.
Laohawetwanit T, 2021 [52]	Thammasat University, Thailand	Pathology, second year medical students	Observational case–control study	29	PathPresenter	There was a significant improvement between student pre-test scores and post-test scores. VM was viewed as a preferred learning modality, mainly because of its portability, satisfactory quality of images, permitting learning in less time, and stimulating cooperation between students while improving interaction with teachers.
Uraiby H, 2021 [53]	University Hospitals of Leicester, UK	Histopathology, medical students	Cross-sectional surveys	90	VM software-Philips Xplore, WSI scanner-Hamamatsu NanoZoomer S210	Study showed a significant improvement in interest, confidence and competence in histopathology. The mean performance scores were significantly increased.
Ali SAA, 2020 [54]	King Khalid University, Saudi Arabia	Histology, dental students	Cross-sectional surveys	129	Not specified	Majority students reported that using VM for practical training sessions makes the oral histology course easier and more interesting.
Samueli B, 2020 [22]	Ben Gurion University of the Negev, Israel	Pathology, medical students	Cross-sectional surveys	59	VM software-CaseViewer (3DHistech, Budapest) and Aperio ImageScope (Leica, Illinois). WSI scanner-Pannoramic MIDI automated digital slide scanner (3DHistech, Budapest).	Study reported an overall favorable response on questions relating to course interest and improvement in understanding of the covered diseases. The most significant disadvantage was technical challenges in accessing the slides.
Parker EU, 2020 [55]	University of Washington School of Medicine, USA	Pathology, medical students	Structured questionnaire survey	70	PathPresenter	The study reported an overwhelmingly positive result regarding understanding of pathology concepts as well as attitudes toward pathology.
Dennis JF, 2020 [56]	Kansas City University, USA	Histology and pathology, medical students	Cross-sectional surveys	200	Virtual Microscopy Database (VMD)	VM use improved student attitudes towards histology content and had a positive impact on student-faculty rapport. Students self-reporting an increased comfortability and understanding with differential diagnosis suggested a strengthening of self-efficacy skills.
Bacha D, 2020 [57]	University of Tunis El Manar, Tunisia	Pathology, medical students	Observational cohort study	45	Not specified	This study reported that performance of the VM is comparable to that of the LM. Thus, VM could serve as an alternative tool to LM in teaching students’ general pathological anatomy.
Lee BC, 2020 [58]	National Taiwan University, Taiwan	Histology and pathology, medical and dental students	Observational case–control study	649	EBM Technologies Inc., Taiwan	The study reported a positive effect of the VM platform on laboratory test Grades was associated with prior experience using the VM platform and was synergistic with more interim tests. Both teachers and students agreed that the VM platform enhanced laboratory learning. The incorporation of the VM platform in the context of test-enhanced learning may help more students master microscopic laboratory content.
Amer MG, 2020 [59]	Taif University, Saudi Arabia	Histology, medical students	Cross-sectional surveys	166	VM software-Aperio’s ImageScope. WSI scanner-Aperio AT2 High Volume (Leica Biosystems).	The study used VM during online objective structural practical examination (OSPE) of 3rd year medical students. The net students feedback was positive and the students recorded the easy image access at any time and place with VM as the most distinctive feature.
Romeike BFM, 2019 [60]	Jena University Hospital, Germany	Histopathology, medical students	Observational case–control study	140	Not specified	This study reported impact of VM use in collaborative “buzz groups’ and showed an overall improvement of the histopathological competencies. The course also increased the appreciation of students for histopathology.
King TS, 2019 [61]	UT-Health, San Antonio, USA	Histology and pathology, medical students	Observational case–control study	220	VM software-Biolucida (MicroBrightField Bioscience). WSI scanner-BLiSSTM-200 (MicroBrightField Bioscience)	The study concluded that VM promoted understanding and encouraged discussion of the topics covered during the week and that group members worked well together and contributed to the completion of the portfolios. Performances on the Histology and Cell Biology and Pathology sections on the United States Medical Licensing Examination (USMLE) remained consistent and in line with national averages.
Husmann PR, 2019 [62]	Indiana University School of Medicine, Indiana, USA	Anatomy, medical students	Cross-sectional surveys	426	Bacus Laboratories (Olympus, 2008)	Statistically significant positive correlations were found with use of VM suggesting that increased use of these resources was more common in students with higher exam scores in the class.
Yohannan DG, 2019 [63]	Government Medical College, Thiruvananthapuram, India	Histopathology, first year medical students	Nonrandomized controlled trial with a crossover design	200	VM software-Aperio’s ImageScope	Majority students agreed that VM made them understand histology better than LM. Almost 90% students agreed that they preferred VM for viewing a histology slide. A paired *t* test indicated that the histology knowledge of the students of both control and test groups significantly improved.
Chen CP, 2019 [64]	University of Pittsburgh School of Medicine, USA	Pathology, medical students	Observational case–control study	123 control group and 164 test group	Tutor (Philips Pathology, Amsterdam, Netherlands), formerly PathXL	The majority students responded positively that the test questions improved their understanding of pediatric diseases (75%) and test questions were helpful in assessing their knowledge of the pediatric pathology (90%), and relative ease of use for the Tutor program (80%).
Nauhria S, 2019 [20]	Windsor University School of Medicine, St. Kitts and Nevis	Pathology, second year medical students	Randomized crossover control study	152	VM software-Aperio’s ImageScope. WSI-IOWA Virtual Slide Box	A majority (83%) of the students preferred to use VM over LM. Students who used VM scored significantly higher in the crossover study compared to those who used LM. This study concluded that using VM to learn histopathology significantly increased student learning and performance compared to using LM.
Tauber Z, 2019 [65]	Palacky University, Czech Republic	Histology, dentistry and general medicine students	Observational case–control study	82 dentistry and 126 general medicine students	Not specified	This study reported that a combination of both electronic materials (VM) and textbooks was commonly used by students with electronic resources being used regularly by the majority of students. No statistically relevant differences were found between the approaches of dentistry versus general medicine students. Cooperation amongst students for individual presentations was seen to be beneficial by a majority of dentistry students.
Felszeghy S, 2019 [66]	University of Eastern Finland, Finland	Histology, medical and dental students	Cross-sectional surveys	160	Whole-slide imaging platform (Aiforia, Fimmic Oy, Finland).	In the open-ended survey, most students viewed collaborative team- and gamification-based learning positively.
Yazid F, 2019 [67]	Universiti Kebangsaan Malaysia, Malaysia	Oral pathology, fourth year dentistry students	Observational case–control study	53	VM software-OlyVIA viewer. WSI-Precipoint M8 microscopescanner.	A majority of students preferred VM over LM and agreed that DM was effective for the course purpose. For the diagnosis exercise, all participants managed to answer correctly using VM compared to LM. Thus, indicating that VM should certainly be integrated as a teaching tool to enhance the learning process within the dental curriculum.

**Table 2:** Summary of quality assessment of cohort studies using NOS scale.

	Selection							Comparability		Outcomes				
Author, Year	Representation of Sample		Selection of the Non-Exposed Cohort	Ascertainment of Exposure			Demonstration That Outcome of Interest was not Present at Start of Study	Comparability of Cohorts on the Basis of the Design or Analysis Controlled for Confounders		Assessment of Outcome		Was Follow-up Long Enough for Outcomes to Occur	Adequacy of Follow-up of Cohorts	
	Truly representative	Somewhat representative	Drawn from the same community as the exposed cohort	Drawn from a different source	Secure record (e.g., surgical record)	Structured interview	Yes	The study controls for age, sex and marital status	Study controls for other factors	Independent blind assessment	Record linkage	Yes	Complete follow up- all subject accounted for	Subjects lost to follow-up unlikely to introduce bias; number lost less than or equal to 20% or description of those lost suggested no different from those followed
Darici D, 2021 [40]	*		*		*		*					*		*
Tauber Z, 2021 [42]		*		*			*					*	*	
Somera dos Santos F, 2021 [44]	*		*				*		*	*		*	*	
Cruz M, 2021 [45]		*		*			*	*	*	*		*	*	
Sharma R, 2021 [46]		*					*			*		*	*	
Liu Q, 2021 [49]	*		*				*		*	*		*	*	
Lee BC, 2020 [58]	*		*			*	*		*	*		*	*	
Yohannan DG, 2019 [63]		*	*			*	*	*			*	*	*	
Sakthi-Velavan S, 2022 [32]		*	*		*		*				*	*	*	

**Table 3:** Summary of quality assessment of cross-sectional studies using the NOS scale.

Author, Year	Selection						Comparability		Outcome		
	Sample Representation		Sample Size Justified	Non-Respondents	Ascertainment of the Exposure		Important Confounding Factors Controlled	Study Control for any Additional Factors	Outcome Assessment		Statistical Test
	All Subjects/Random Sampling	Non-Random Sampling			Validated Measurement Tool. **	Non-Validated Measurement Tool, but the Tool is Available or Described. *			Independent Blind Assessment/Record Linkage. **	Self-Report *	
Nikas IP, 2021 [36]		*			**				**		*
Chang JYF, 2021 [39]				*				*	**		*
Tanaka KS, 2021 [41]				*				*		*	*
Guiter GE, 2021 [43]				*		*		*			*
White MJ, 2021 [47]		*				*				*	
Yakin M, 2021 [38]		*								*	
Lakhtakia R, 2021 [48]		*								*	
Uraiby H, 2021 [53]						*					
Samueli B, 2020 [22]		*								*	
Bacha D, 2020 [57]		*								*	*
Amer M, 2020 [59]		*		*	**			*	**		*
Ali SAA, 2020 [54]	*					*				*	*
Romeike BFM, 2019 [60]		*	*	*	**			*	**		*
Tauber Z, 2019 [65]		*				*				*	
King TS, 2019 [61]				*		*			**		*
Husmann PR, 2019 [62]											
Felszeghy S, 2019 [66]		*		*		*	*		**		
Manou E, 2021 [51]				*						*	
Yazid F, 2019 [67]											
Laohawetwanit T, 2021 [52]				*		*		*	**		

**Table 4:** The summary of quality assessment of randomized controlled studies using NOS scale.

Author, Year	Selection				Comparability		Exposure		
	Adequate Case Definition	Case Representativeness	Selection of Control	Definition of Control	Important Study Control	Study Controls for any Additional Factors	Ascertainment of Exposure	Same Method of Ascertainment for Cases and Controls	Non-Response Rate
Nauhria S, 2019 [20]	*	*	*	*	*		*	*	*
Simok, A.A. 2021 [50]	*	*	*	*		*	*	*	*
Ahmed S, 2022 [33]		*	*		*		*	*	*

**Table 5:** Summary of quality assessment of case–control studies using the NOS scale.

Author, Year	Selection				Comparability		Exposure		
	Adequate Case Definition (Yes, with Independent Validation)	Case representativeness (Consecutive or Obviously Representative Series of Cases)	Selection of Control (Community Controls)	Definition of Control: no History of Disease (Endpoint)	Important Study Control	Study Control for any Additional Factors	Ascertainment of Exposure	Same Method of Ascertainment for Cases and Controls	Non-Response Rate
Waugh S, 2022 [31]	*	*	*				*	*	*
Zhong Y, 2021 [37]		*	*	*	*		*	*	
Chen CP, 2019 [64]	*		*			*	*	*	*
Sakthi-Velavan S, 2022 [32]									
Yazid F, 2019 [67]	*			*			*	*	*
Qing J, 2022 [34]	*	*	*		*		*	*	*
Francis DV, 2022 [35]	*	*	*	*		*	*	*	*

## Supplementary Materials

The following supporting information can be downloaded at https://www.mdpi.com/article/10.3390/diagnostics13030558/s1. Table S1: Themes depicting advantages/disadvantages of VM in comparison to LM; File S1: Newcastle—Ottawa Quality assessment scale case control studies; File S2: Thresholds for converting the Newcastle-Ottawa scales to AHRQ standards.
