# Supplementary material for: Virtual Versus Light Microscopy Usage among Students: A Systematic Review and Meta-Analytic Evidence in Medical Education
Source: Diagnostics (Basel). 2023 Feb 2;13(3):558. doi: 10.3390/diagnostics13030558 (PMC9914930; doi:10.3390/diagnostics13030558)
Supplement: Supplementary file 1 [file diagnostics-13-00558-s001.zip › diagnostics-1815413-File S1-S2.pdf]

Supplementary File S1: NEWCASTLE - OTTAWA QUALITY ASSESSMENT SCALE CASE CONTROL STUDIES

Note: A study can be awarded a maximum of one star for each numbered item within the Selection and Exposure categories. A maximum of two stars can be given for Comparability.

Selection

1) Is the case definition adequate?
   a) yes, with independent validation ✵
   b) yes, eg record linkage or based on self reports
   c) no description

2) Representativeness of the cases
   a) consecutive or obviously representative series of cases ✵
   b) potential for selection biases or not stated

3) Selection of Controls
   a) community controls ✵
   b) hospital controls
   c) no description

4) Definition of Controls
   a) no history of disease (endpoint) ✵
   b) no description of source

Comparability

1) Comparability of cases and controls on the basis of the design or analysis
   a) study controls for _______________ (Select the most important factor.) ✵
   b) study controls for any additional factor ✵ (This criteria could be modified to indicate specific control for a second important factor.)

Exposure

1) Ascertainment of exposure
   a) secure record (eg surgical records) ✵
   b) structured interview where blind to case/control status ✵
   c) interview not blinded to case/control status
   d) written self report or medical record only
   e) no description

2) Same method of ascertainment for cases and controls
   a) yes ✵
   b) no

3) Non-Response rate
   a) same rate for both groups ✵
   b) non respondents described
   c) rate different and no designation

NEWCASTLE - OTTAWA QUALITY ASSESSMENT SCALE
COHORT STUDIES

<u>Note</u>: A study can be awarded a maximum of one star for each numbered item within the Selection and Outcome categories. A maximum of two stars can be given for Comparability

Selection

1) <u>Representativeness of the exposed cohort</u>
   a) truly representative of the average _______________ (describe) in the community ✵
   b) somewhat representative of the average ______________ in the community ✵
   c) selected group of users eg nurses, volunteers
   d) no description of the derivation of the cohort

2) <u>Selection of the non exposed cohort</u>
   a) drawn from the same community as the exposed cohort ✵
   b) drawn from a different source
   c) no description of the derivation of the non exposed cohort

3) <u>Ascertainment of exposure</u>
   a) secure record (eg surgical records) ✵
   b) structured interview ✵
   c) written self report
   d) no description

4) <u>Demonstration that outcome of interest was not present at start of study</u>
   a) yes ✵
   b) no

Comparability

1) <u>Comparability of cohorts on the basis of the design or analysis</u>
   a) study controls for _____________ (select the most important factor) ✵
   b) study controls for any additional factor ✵ (This criteria could be modified to indicate specific control for a second important factor.)

Outcome

1) <u>Assessment of outcome</u>
   a) independent blind assessment ✵
   b) record linkage ✵
   c) self report
   d) no description

2) <u>Was follow-up long enough for outcomes to occur</u>
   a) yes (select an adequate follow up period for outcome of interest) ✵
   b) no

3) <u>Adequacy of follow up of cohorts</u>
   a) complete follow up - all subjects accounted for ✵
   b) subjects lost to follow up unlikely to introduce bias - small number lost - > ____ % (select an adequate %) follow up, or description provided of those lost) ✵
   c) follow up rate < ____% (select an adequate %) and no description of those lost
   d) no statement

Newcastle-Ottawa Scale adapted for cross-sectional studies

Selection: (Maximum 5 stars)

1) Representativeness of the sample:

a) Truly representative of the average in the target population. * (all subjects or random sampling)

b) Somewhat representative of the average in the target population. * (non-random sampling)

c) Selected group of users.

d) No description of the sampling strategy.

2) Sample size:

a) Justified and satisfactory. *

b) Not justified.

3) Non-respondents:

a) Comparability between respondents and non-respondents characteristics is established, and the response rate is satisfactory. *

b) The response rate is unsatisfactory, or the comparability between respondents and non-respondents is unsatisfactory.

c) No description of the response rate or the characteristics of the responders and the non-responders.

4) Ascertainment of the exposure (risk factor):

a) Validated measurement tool. **

b) Non-validated measurement tool, but the tool is available or described.*

c) No description of the measurement tool.

Comparability: (Maximum 2 stars)

1) The subjects in different outcome groups are comparable, based on the study design or analysis. Confounding factors are controlled.

a) The study controls for the most important factor (select one). *

b) The study control for any additional factor. *

Outcome: (Maximum 3 stars)

1) Assessment of the outcome:

a) Independent blind assessment. **

b) Record linkage. **

c) Self report. *

d) No description.

2) Statistical test:

a) The statistical test used to analyze the data is clearly described and appropriate, and the measurement of the association is presented, including confidence intervals and the probability level (p value). *

b) The statistical test is not appropriate, not described or incomplete.

This scale has been adapted from the Newcastle-Ottawa Quality Assessment Scale for cohort studies to perform a quality assessment of cross-sectional studies for the systematic review, "**Bullying and health related quality of life among adolescents- a systematic review**".

NEWCASTLE - OTTAWA QUALITY ASSESSMENT SCALE: RANDOMIZED CONTROLLED TRIAL

Note: A study can be awarded a maximum of one star (*) for each numbered item within the Selection and Exposure categories. A maximum of two stars can be given for Comparability.

Selection

1) Is the case definition adequate?
    a) yes, with independent validation *
    b) yes, e.g., record linkage or based on self reports
    c) no description
2) Representativeness of the cases
    a) consecutive or obviously representative series of cases *
    b) potential for selection biases or not stated
3) Selection of Controls
    a) community controls *
    b) hospital controls
    c) no description
4) Definition of Controls
    a) no history of disease (endpoint) *
    b) no description of source

Comparability

1) Comparability of cases and controls on the basis of the design or analysis
    a) study controls for _______________ (Select the most important factor.) *
    b) study controls for any additional factor * (This criteria could be modified to indicate specific control for a second important factor.)

Exposure

1) Ascertainment of exposure
    a) secure record (eg surgical records) *
    b) structured interview where blind to case/control status *
    c) interview not blinded to case/control status
    d) written self report or medical record only
    e) no description
2) Same method of ascertainment for cases and controls
    a) yes *
    b) no
3) Non-Response rate
    a) same rate for both groups *
    b) non respondents described
    c) rate different and no designation

Supplementary File S2:

Thresholds for converting the Newcastle-Ottawa scales to AHRQ standards (good, fair, and poor):

Good quality: 3 or 4 stars in selection domain AND 1 or 2 stars in comparability domain AND 2 or 3 stars in outcome/exposure domain

Fair quality: 2 stars in selection domain AND 1 or 2 stars in comparability domain AND 2 or 3 stars in outcome/exposure domain

Poor quality: 0 or 1 star in selection domain OR 0 stars in comparability domain OR 0 or 1 stars in outcome/exposure domain
