# Supplementary material for: Virtual Versus Light Microscopy Usage among Students: A Systematic Review and Meta-Analytic Evidence in Medical Education
Source: Diagnostics (Basel). 2023 Feb 2;13(3):558. doi: 10.3390/diagnostics13030558 (PMC9914930; doi:10.3390/diagnostics13030558)
Supplement: Supplementary file 1 [file diagnostics-13-00558-s001.zip › diagnostics-1815413-Table S1.pdf]

Supplementary Table S1: Themes depicting advantages/disadvantages of VM in comparison to LM

Themes			References
**Advantages**	Ease of use	Availability of free online resources	31, 36, 38, 42, 43, 20
		Accessibility to slides outside of the classroom	35, 44, 47, 48, 49, 52, 53, 54, 59, 64, 67
		Efficiency and feasibility	34, 40, 41, 44, 48, 54, 20, 67
		Better image quality, no loss of quality/slides over time	34, 35, 36, 42, 47, 53, 58, 60, 67
		Ability to annotate slides	35, 20
	Improved collaboration	Improved student faculty rapport and collaboration	33, 34, 42, 45, 51, 52, 56, 60
		Better cooperation and participation amongst students	31, 32, 33, 34, 36, 43, 52, 57, 60, 61, 20, 66, 67
	Exam preparation	Better time management	32, 52
		Better source for exam preparation	32, 38, 56
		Higher student confidence/satisfaction	32, 38, 53
		Promotion of self-directed learning	31, 35, 38, 41, 48, 58, 59, 64
	Academic performance	USMLE scores correlation	61
		Significantly improved academic test scores	31, 32, 36, 37, 38, 46, 48, 50, 52, 53, 58, 62, 63, 20
		Better for remote/online learning	32, 40, 41
		Improved medical knowledge of the subject	34, 38, 44, 45, 46, 48, 22, 55, 58, 61, 63, 64, 67
		Improved diagnostic and practical skill development during laboratory sessions	33, 39, 48, 53, 56, 5, 60, 67
		Improved class attendance	47, 48, 54
	Faculty impact	Higher level of Faculty/teacher satisfaction	35, 42, 57, 59, 20
		Cost effectiveness/ time effectiveness	31, 32, 33, 35, 36, 42, 57, 20, 67
	Student perceptions and acceptability	Overall positive acceptance for VM/ higher student satisfaction for VM based teaching	36, 37, 39, 41, 47 49, 50, 22, 59, 64, 20, 67

		VM or the combination of VM together with the examination of glass mounted specimens by microscope	42
		VM use increased the level of subject interest in students	35, 39, 53, 54, 22, 55, 60, 66
		VM reported as the preferred method for learning	42, 52, 63, 67
		Positive perception and improved student satisfaction	34, 36, 55
		The knowledge acquisition on using VM was comparable to the LM	33, 42, 44, 50, 57, 61, 64, 20, 65
**Disadvantages**			
	Technical implication	Technical/internet issues while accessing the slides	33, 38, 44, 22, 57
	Absence of interaction	Impaired social connections	41, 44, 22
		Absence of specimen handling experience/training issues	41, 44, 67
		Inadequate feedback from the expert/faculty	41, 44
	No academic score improvement		65
		Further research needed to enhance understanding impact of such technologies and for educational policy formulation	51, 52
